# Supplementary material for: Multi‐dataset identification of innovative feature genes and molecular mechanisms in keratoconus
Source: J Cell Mol Med. 2024 Sep 19;28(18):e70079. doi: 10.1111/jcmm.70079 (PMC11412914; doi:10.1111/jcmm.70079)
Supplement: Supplementary file 2 — Material S2. [file JCMM-28-e70079-s002.zip › 7.GSEA analysis/4.MYRF/KEGG_RNA_DEGRADATION.html]

Details for gene set KEGG\_RNA\_DEGRADATION[GSEA]

|  || Dataset | MYRF.group.cls#h\_versus\_l |
| Phenotype | group.cls#h\_versus\_l |
| Upregulated in class | h |
| GeneSet | KEGG\_RNA\_DEGRADATION |
| Enrichment Score (ES) | 0.19461906 |
| Normalized Enrichment Score (NES) | 1.0073885 |
| Nominal p-value | 0.4969072 |
| FDR q-value | 1.0 |
| FWER p-Value | 1.0 |
Table: GSEA Results Summary

  

Fig 1: Enrichment plot: KEGG\_RNA\_DEGRADATION      
 Profile of the Running ES Score & Positions of GeneSet Members on the Rank Ordered List

  

| SYMBOL | TITLE | RANK IN GENE LIST | RANK METRIC SCORE | RUNNING ES | CORE ENRICHMENT || 1 | DIS3 | na | 1969 | 0.359 | 0.0672 | Yes |
| 2 | CNOT1 | na | 6908 | 0.166 | -0.0176 | Yes |
| 3 | EXOSC6 | na | 7516 | 0.150 | 0.0168 | Yes |
| 4 | DDX6 | na | 10279 | 0.091 | -0.0314 | Yes |
| 5 | CNOT6 | na | 10658 | 0.084 | -0.0134 | Yes |
| 6 | CNOT6L | na | 10801 | 0.082 | 0.0107 | Yes |
| 7 | DCPS | na | 11213 | 0.075 | 0.0247 | Yes |
| 8 | DCP2 | na | 11859 | 0.065 | 0.0287 | Yes |
| 9 | PATL1 | na | 12042 | 0.063 | 0.0453 | Yes |
| 10 | EXOSC3 | na | 12717 | 0.054 | 0.0444 | Yes |
| 11 | PAPOLB | na | 13171 | 0.048 | 0.0478 | Yes |
| 12 | PAPOLA | na | 13541 | 0.043 | 0.0522 | Yes |
| 13 | ZCCHC7 | na | 13609 | 0.043 | 0.0650 | Yes |
| 14 | EXOSC4 | na | 13690 | 0.042 | 0.0771 | Yes |
| 15 | ENO1 | na | 14230 | 0.035 | 0.0737 | Yes |
| 16 | CNOT10 | na | 15688 | 0.021 | 0.0389 | Yes |
| 17 | EXOSC1 | na | 16467 | 0.013 | 0.0210 | Yes |
| 18 | PARN | na | 16546 | 0.013 | 0.0231 | Yes |
| 19 | CNOT3 | na | 16785 | 0.010 | 0.0198 | Yes |
| 20 | CNOT2 | na | 16942 | 0.009 | 0.0185 | Yes |
| 21 | XRN1 | na | 17275 | 0.006 | 0.0110 | Yes |
| 22 | LSM3 | na | 17289 | 0.006 | 0.0127 | Yes |
| 23 | DCP1B | na | 17357 | 0.005 | 0.0127 | Yes |
| 24 | EXOSC9 | na | 17729 | 0.002 | 0.0027 | Yes |
| 25 | LSM7 | na | 17751 | 0.002 | 0.0027 | Yes |
| 26 | LSM1 | na | 18158 | -0.002 | -0.0083 | Yes |
| 27 | EXOSC2 | na | 18173 | -0.002 | -0.0079 | Yes |
| 28 | PAPOLG | na | 18470 | -0.005 | -0.0148 | Yes |
| 29 | EXOSC8 | na | 18746 | -0.008 | -0.0201 | Yes |
| 30 | EDC3 | na | 19136 | -0.012 | -0.0271 | Yes |
| 31 | LSM5 | na | 19446 | -0.016 | -0.0307 | Yes |
| 32 | DCP1A | na | 19752 | -0.019 | -0.0330 | Yes |
| 33 | LSM8 | na | 20017 | -0.022 | -0.0330 | Yes |
| 34 | ENO3 | na | 20250 | -0.025 | -0.0311 | Yes |
| 35 | CNOT7 | na | 20469 | -0.027 | -0.0280 | Yes |
| 36 | HSPD1 | na | 20547 | -0.028 | -0.0205 | Yes |
| 37 | C1D | na | 20605 | -0.029 | -0.0121 | Yes |
| 38 | LSM4 | na | 20872 | -0.032 | -0.0087 | Yes |
| 39 | XRN2 | na | 22070 | -0.047 | -0.0271 | Yes |
| 40 | LSM2 | na | 22108 | -0.047 | -0.0119 | Yes |
| 41 | PNPT1 | na | 22586 | -0.053 | -0.0072 | Yes |
| 42 | LSM6 | na | 22613 | -0.054 | 0.0106 | Yes |
| 43 | EXOSC10 | na | 23358 | -0.065 | 0.0116 | Yes |
| 44 | EDC4 | na | 23754 | -0.072 | 0.0250 | Yes |
| 45 | EXOSC7 | na | 23976 | -0.075 | 0.0447 | Yes |
| 46 | HSPA9 | na | 24642 | -0.087 | 0.0555 | Yes |
| 47 | CNOT4 | na | 26249 | -0.118 | 0.0501 | Yes |
| 48 | EXOSC5 | na | 26301 | -0.119 | 0.0898 | Yes |
| 49 | MPHOSPH6 | na | 26570 | -0.125 | 0.1252 | Yes |
| 50 | ENO2 | na | 27917 | -0.158 | 0.1410 | Yes |
| 51 | CNOT8 | na | 27979 | -0.160 | 0.1946 | Yes |
Table: GSEA details [plain text format]

  

Fig 2: KEGG\_RNA\_DEGRADATION      
 Blue-Pink O' Gram in the Space of the Analyzed GeneSet

  

Fig 3: KEGG\_RNA\_DEGRADATION: Random ES distribution      
 Gene set null distribution of ES for **KEGG\_RNA\_DEGRADATION**
